# An IPTG Inducible Conditional Expression System for Mycobacteria

**DOI:** 10.1371/journal.pone.0134562

**Published:** 2015-08-06

**Authors:** Sudha Ravishankar, Anisha Ambady, Haripriya Ramu, Naina Vinay Mudugal, Ragadeepthi Tunduguru, Anand Anbarasu, Umender K. Sharma, Vasan K. Sambandamurthy, Sudha Ramaiah

**Affiliations:** 1 AstraZeneca India Pvt Ltd, Bellary Road, Hebbal, Bengaluru, Karnataka, India; 2 School of Biosciences & Technology, VIT University, Vellore, Tamil Nadu, India; University of Delhi, INDIA

## Abstract

Conditional expression strains serve as a valuable tool to study the essentiality and to establish the vulnerability of a target under investigation in a drug discovery program. While essentiality implies an absolute requirement of a target function, vulnerability provides valuable information on the extent to which a target function needs to be depleted to achieve bacterial growth inhibition followed by cell death. The critical feature of an ideal conditional expression system is its ability to tightly regulate gene expression to achieve the full spectrum spanning from a high level of expression in order to support growth and near zero level of expression to mimic conditions of gene knockout. A number of bacterial conditional expression systems have been reported for use in mycobacteria. The utility of an isopropylthiogalactoside (IPTG) inducible system in mycobacteria has been reported for protein overexpression and anti-sense gene expression from a replicating multi-copy plasmid. Herein, we report the development of a versatile set of non-replicating IPTG inducible vectors for mycobacteria which can be used for generation of conditional expression strains through homologous recombination. The role of a single *lac* operator versus a double *lac* operator to regulate gene expression was evaluated by monitoring the expression levels of β-galactosidase in *Mycobacterium smegmatis*. These studies indicated a significant level of leaky expression from the vector with a single *lac* operator but none from the vector with double *lac* operator. The significance of the double *lac* operator vector for target validation was established by monitoring the growth kinetics of an *inhA*, *a rpoB* and a *ftsZ* conditional expression strain grown in the presence of different concentrations of IPTG. The utility of this inducible system in identifying target specific inhibitors was established by screening a focussed library of small molecules using an *inhA* and a *rpoB* conditional expression strain.

## Introduction

The process of target based drug discovery and development is laborious, expensive, and time consuming [1]. The targets chosen for a target based drug discovery program have to be linked to the disease onset and progression. Additionally, in the anti-bacterial field, these targets should ideally have no human homologs or be selective enough to minimize mechanism based toxicity issues [1]. Due to the emergence of antibiotic resistance, demand for identifying novel antibacterial targets is on the rise [2, 3]. Whole genome sequencing of many bacterial pathogens has unveiled numerous metabolic pathways and their critical enzymes to be exploited for a drug discovery program [4]. However, the relevance of these ‘new’ targets to the disease biology both under *in vitro* and *in vivo* growth conditions needs to be established before embarking on a massive drug screening campaign [1]. Target validation is therefore a critical step during the drug discovery phase to assess the essentiality of a chosen target gene for the survival of the target pathogen. As a general practice, the essentiality of a selected target is determined by generating a knockout strain of the respective gene and studying the effect on survival of the specific bacterial pathogen [3]. Although, a knockout strain provides information regarding the absolute essentiality of a target gene, such a strain cannot be used to ascertain the target requirement for the survival of the pathogen under various physiological conditions. On the contrary, a conditional expression or knockdown (KD) strain generated using regulated inducible expression system enables the testing of essentiality under both *in vitro* and *in vivo* growth conditions [5]. The ability to modulate gene expression by varying the inducer concentration in the growth environment allows the use of conditional expression strains to study the effect of target depletion under a variety of physiological conditions and therefore the target’s relevance to disease biology. A number of such inducible expression systems have been reported for use in bacteria including mycobacteria [6–21]. While many of them were used to establish gene essentiality *in vitro*, some of them have been employed to assess gene essentiality under both *in vitro* and *in vivo* growth conditions. A few of these systems employed antibiotics such as doxycycline [6–7, 14–19] or pristinamycin [8, 20] as inducer. Such a system could be experimentally challenging as these molecules have inherent antibacterial activity. Although, there are several reports of the successful application of a *tet* system for target evaluation in *Mycobacterium tuberculosis* under both *in vitro* and *in vivo* conditions [6–7, 14–19], there is always a distinct possibility that the inducer concentration required to achieve sufficient expression for growth could reach non-permissible levels resulting in growth arrest. The concentration of inducer required for modulating gene expression and thereby the phenotype is likely to depend on the target gene. In one of the earlier published work on *M*. *tuberculosis pimA* knock down study [18], doxycycline was used at levels (0.25–0.5 μg/ml) close to its antibacterial concentration (1–2 μg/ml). Similarly, the peptide component of pristinamycin was purified to safely use it as inducer [8, 20] because of its less potent MIC (≥2 μg/ml) for *M*. *tuberculosis* compared to the MIC of antibiotic pristinamycin (~0.1 μg/ml). There are a few inducible systems which have used non-antibiotic metabolites like arabinose, acetamide, IPTG or theophylline as inducer. However, except for a few, most of the studies involving these systems have investigated target validation only under *in vitro* growth conditions [6–9, 11–14, 15–18].

IPTG inducible system has been widely used for recombinant protein expression in *E*. *coli*. Its components like *lac* promoter, *lac* operator, *lac* repressor and their variants have been employed along with T7 or other promoters in various combinations to achieve better regulation in gene expression [22]. While the use of an IPTG system for conditional expression has been established under both *in vitro* and *in vivo* conditions for *S*. *aureus* [10], Kaur *et al* have demonstrated the value of the IPTG system for conditional expression in mycobacteria under *in vitro* growth conditions [11]. Due to the absence of a *lac* operon and hence a *lac* permease, IPTG is thought to diffuse through the mycobacterial membrane thereby enabling the expression of a gene cloned downstream of a Plac or Ptrc promoter in a dose-dependent manner. The successful use of an IPTG inducible system in mycobacteria for employing a cell based screening assay to identify protein splicing inhibitors [23] and to distinguish metabolically active *M*. *tuberculosis* cells from those which are inactive upon macrophage infection through expression of GFP [24] confirms the possibility of obtaining an IPTG dose-dependent expression of proteins in mycobacteria. However, the antisense expression system reported by Kaur *et al*. seems to reach a saturation effect at 10 μM IPTG with no dose-dependent phenotype observed in all of the survival kinetics studies performed [11]. This could be due to the accumulation of anti-sense message expressed from a multi-copy plasmid prior to induction with IPTG. The presence of such leaky expression in the anti-sense mediated conditional expression system may result in the inability to obtain recombinants for genes which are very sensitive to the presence of even small quantities of anti-sense transcripts. In the present study, we aimed to develop and evaluate non-replicating IPTG inducible conditional expression vectors which would stably integrate into the host chromosome, thereby enabling reduction in the leaky expression and circumventing the saturation effects observed earlier. A short segment (about 500–600 bp) of a target gene from its 5’ end can be cloned downstream of the Ptrc promoter in this vector and used as a substrate for homologous recombination. A single crossover recombination event following electroporation with such a recombinant plasmid would result in integration of the plasmid into the host chromosome with specific insertion of the Ptrc promoter upstream of the gene of interest. The resulting strain would have a single copy of the full length gene under the control of an IPTG inducible promoter enabling a dose-dependent expression of the gene under investigation.

Herein, we report the generation of a non-replicating IPTG inducible conditional expression vector which contains Ptrc promoter along with *lac* operator (*lacO*) and *lac* repressor (*lacI*). The stringency of gene expression from this recombinant vector was analysed using two different *lac* operator systems, a single *lac* operator as reported by Kaur *et al* [11] and a double *lac* operator as reported by Tobbell *et al* [25]. IPTG dose-dependent expression of *lacZ*, *inhA*, *rpoB* and *ftsZ* were tested in *M*. *smegmatis* mc^2^155 to validate the robustness of the system. Furthermore, the hypersensitivity phenotype of the *inhA* and *rpoB* conditional expression strains to isoniazid and rifampicin respectively, led us to exploit these recombinant strains to identify target specific inhibitors via cell based screening.

## Materials and Methods

### Bacterial strains, media, chemicals and reagents

Bacterial strains used in this study are listed in Table 1. For growing *E*. *coli*, Luria Bertani (LB) broth and LB agar was used and supplemented with appropriate antibiotic as required. 7H9 broth supplemented with 0.2% glycerol (v/v), 0.05% tween 80 (w/v) and 7H11 were used for the growth of mycobacteria with the addition of appropriate antibiotics and IPTG as required. Restriction enzymes, 1kb DNA ladder were obtained from New England Biolabs, Hygromycin B was obtained from Roche, IPTG was purchased from SIGMA, Hybond membrane and chemiluminescence Western blot kits were from GE Healthcare, 0.1 mm Zirconia beads and Mini bead beater were from Biospec products. Bradford reagent was obtained from Pierce Biotechnology Inc., and protease inhibitor cocktail was from Roche. Polyclonal antibodies were custom prepared either at Bangalore Genei India Pvt. Ltd. or Abexome Biosciences.

**Table 1:** List of plasmids and strains used in this study.

Plasmids/ Strains	Reference	Details
pAZI9018b	[11 *]	Replicating vector with Ptrc promoter
pT73.3	[25 *]	Replicating vector with T7 promoter and two palindromic *lac* operators
pAZI272	[26 *]	Non-replicating plasmid for gene expression in mycobacteria with a hsp60 promoter
pMV261	[27 *]	Replicating plasmid for gene expression in mycobacteria with a hsp60 promoter
pAZI0261	This study	Conditional expression vector with Ptrc promoter and single *lac* operator
pAZI9452	This study	Conditional expression vector with Ptrc promoter and two *lac* operators
pAZI0233	This study	Conditional expression plasmid of *lacZ* derived from pAZI9018b with attP-*int*.
pBAN0195	This study	Intermediate vector during preparation of pBAN0196 from pAZI9452 with *lacZ* cloned
pBAN0196	This study	Conditional expression plasmid of *lacZ* derived from pAZI9452 with attP-*int* cloned.
pAZI9464	This study	Conditional expression plasmid of *M*. *smegmatis inhA* in pAZI9452
pAZI9466	This study	Conditional expression plasmid of *M*. *smegmatis rpoB* in pAZI9452
pAZI9470	This study	Conditional expression plasmid of *M*. *smegmatis ftsZ* in pAZI9452
pAZI9472	This study	Conditional expression plasmid of *M*. *smegmatis rpoB* in pAZI0261
pAZI9474	This study	*M*. *smegmatis hemH* cloned into pAZI272 for complementation in *inhA*/KD/DO strain.
pAZI9476	This study	*M*. *smegmatis inhA gene cloned in pMV261 vector*
*lacZ*/KD/SO	This study	Conditional expression strain of *lacZ* derived using pAZI0233
*lacZ*/KD/DO	This study	Conditional expression strain of *lacZ* derived using pBAN0196
*inhA*/KD/DO	This study	Conditional expression strain of *M*. *smegmatis inhA* derived using pAZI9464
*rpoB*/KD/DO	This study	Conditional expression strain of *M*. *smegmatis rpoB* derived using pAZI9466
*ftsZ*/KD/DO	This study	Conditional expression strain of *M*. *smegmatis ftsZ* derived using pAZI9470

*References

All the primers (oligonucleotides) used for amplification of genes using polymerase chain reaction (PCR), screening of recombinant plasmids and recombinant strains are listed in S1 Table. All the recombinant strains generated as part of this study and plasmids used in this study are listed in Table 1.

### Generation of non-replicating conditional expression vectors

A vector with single *lac* operator (pAZI0261) was generated using pAZI9018b [11] as the parent plasmid. The *lacZ* gene was excised from pAZI9018b upon digestion with BamHI and NdeI, the ends were filled with Klenow fragment followed by ligation. The resulting plasmid was rendered non-replicating for mycobacteria by excising the pAL5000 origin (mycobacterial origin of replication) *via* digestion at NheI and ApaI sites followed by ligation after end filling to generate pAZI0261.

A vector with two *lac* operators (pAZI9452) was generated using pT73.3 [25] as the parent plasmid. This vector has a T7 promoter flanked on either side by palindromic *lac* operator sequences enabling tight regulation of gene expression. The *tetA-tetR* segment was replaced by digesting the vector with BseRI followed by ligating the vector fragment with end filled hygromycin resistance gene excised from pAZI9018b by NcoI-NruI digestion. The resulting plasmid (pAZI9451) was digested with NcoI-XbaI to remove the T7 promoter with its two *lac* operators. SynPro (S1 Table), a synthetic gene coding for Ptrc promoter with two *lac* operators, one on either side of the promoter sequence, and having NcoI and XbaI at the 5’ and 3’ end respectively, was cloned into pAZI9451.

### Generation of *lacZ* conditional expression plasmids

Mycobacteria do not have a *lacZ* gene and hence integration of this gene into the chromosome was mediated through the *attP-int* system. Recombinant conditional expression plasmids with *lacZ* gene were generated as described below.

#### pBAN0196

As a first step, a full length *lacZ* gene was cloned as a blunt ended PCR amplified DNA fragment into KpnI digested and T4 DNA polymerase treated pAZI9452 to generate pBAN0195. The *attP-int* sequence released as an XbaI fragment from pAZI272 was cloned into pBAN0195 at SpeI site to generate pBAN0196 (XbaI and SpeI are compatible sites). Recombinant plasmids were screened for an increase in size and the absence of SpeI restriction site.

#### pAZI0233

pAL5000 origin (mycobacterial origin of replication) was excised from pAZI9018b by digestion with NheI and ApaI restriction enzymes. The vector fragment was end filled and ligated to blunt ended fragment of *attP-int* released from pAZI272 with XbaI digestion followed by treatment with Klenow fragment. The recombinant plasmids were screened for the absence of XbaI site.

### Generation of *inhA*, *rpoB* and *ftsZ* conditional expression plasmids

About 600 bps of *inhA*, *rpoB* and *ftsZ* genes from their 5’ ends were amplified using primers listed in S1 Table and cloned into pAZI9452 to generate the conditional expression plasmids pAZI9464, pAZI9466 and pAZI9470 respectively. The truncated *rpoB* gene fragment was also cloned into pAZI0261 to generate pAZI9472. The authenticity of recombinant plasmids were confirmed by PCR and restriction enzyme analysis followed by sequencing of the amplified gene fragments cloned. All recombinant plasmids used in this study are listed in Table 1.

### Generation of *lacZ*, *inhA*, *rpoB* and *ftsZ* conditional expression strains

Conditional expression plasmids of *lacZ*, *inhA*, *rpoB* and *ftsZ* were electroporated into *M*. *smegmatis* mc^2^155. While the *lacZ* transformants were selected on 7H9-agar plates supplemented with 50 μg/ml hygromycin alone, the *inhA*, *rpoB* and *ftsZ* transformants were selected on 7H9-agar plates supplemented with 50 μg/ml hygromycin and 500 μM IPTG. *lacZ* transformants were confirmed by PCR for the presence of the hygromycin resistance gene in their genomic DNA. The bonafide recombinants were denoted as *lacZ*/KD/SO and *lacZ*/KD/DO for knockdown (KD) strains with single operator (SO) and double operator (DO), respectively. The genotype of single cross over recombinants derived from transformation of *M*. *smegmatis* mc^2^155 with *inhA*, *rpoB* and *ftsZ* conditional expression plasmids were confirmed using PCR for the presence of hygromycin resistance gene and additional PCRs to confirm that the genomic organisation of single cross over recombinants, where in the cloned truncated gene fragment (about 600 bps) would be downstream of the native promoter and the full length wild-type gene would be downstream of the Ptrc promoter. The primers used for all the screening work are listed in S1 Table. The resulting strains were denoted as *rpoB*/KD/SO for the strain with a single *lac* operator and *inhA*/KD/DO, *rpoB*/KD/DO, *ftsZ*/KD/DO for the strains with two *lac* operators.

### Generation of an *inhA*/KD/DO strain complemented with *hemH*

A recombinant plasmid harbouring a full length *hemH* gene was cloned downstream of the hsp60 promoter in pAZI272 vector [26], a non-replicating plasmid with *attP-int* sequence for integration into mycobacterial chromosome. The resultant plasmid pAZI9474 was confirmed by restriction analysis and then electroporated into *inhA*/KD/DO recombinant strain. The transformants were selected for resistance to both hygromycin and kanamycin. The resulting recombinant strain was confirmed by PCR amplification of the kanamycin resistance gene present on pAZI9474. This strain referred as *inhA*/KD/DO/*hemH* was used for all *inhA*/KD experiments unless otherwise mentioned.

### PCR Screening of recombinant strains

The screening of recombinant strains in *M*. *smegmatis* was done by PCR using Taq DNA polymerase. Single colonies were picked from plates, resuspended in 50 μl TE (10 mM Tris, 0.1 mM EDTA pH 8.0) and boiled for 20 minutes. 5 μl of the cell lysate was used as a template for amplification using PCR in a 25 μl PCR. The denaturation and extension steps were performed at 94°C and 72°C respectively. The annealing temperature was based on the melting temperatures of the primer pair. The extension time of PCR was based on the length of the PCR product amplified (about 1min / kb). All the primers used for PCR amplification are listed in S1 Table.

### β-galactosidase expression

*lacZ*/KD/SO and *lacZ*/KD/DO strains were grown with 50 μg/ml hygromycin until they reached an optical density (A_600_) of ~1.0. This culture was diluted in 7H9 broth to prepare a master culture containing ~10^6^ CFU/ml to which hygromycin was added to a final concentration of 50 μg/ml and 5-bromo-4-chloro-3-indolyl-β-D-galactopyranoside (X-gal) to a final concentration 40 μg/ml. The master culture was split into many tubes each of which received a different concentration of IPTG. The development of blue color was monitored following 24 hours of growth at 37°C.

### Hemin dependence of *inhA*/KD/DO strain

The *inhA*/KD/DO and *inhA*/KD/DO/*hemH* strains were grown in the presence of 50 μM IPTG and 50 μg/ml hygromycin. The cells were washed three times with fresh culture media to remove the inducer, resuspended in 7H9 broth to be used as an inoculum. Appropriate dilutions of the washed cells were plated on two sets of 7H11 plates, one set supplemented with different concentrations (0, 5, 50 μM) of IPTG alone, a second set supplemented with 40 μg/ml of hemin in addition to IPTG. The plates were incubated at 37°C until colonies appeared. The colony numbers were converted to CFU/ml and a graph was generated to plot the CFU versus IPTG concentration.

### Analysis of IPTG dependence for the growth of *inhA*, *rpoB* and *ftsZ* KD strains

The confirmed KD strains of *inhA*, *ftsZ* and *rpoB* were grown in 7H9 broth supplemented with 50 μg/ml hygromycin and 0.5 mM IPTG till they reached mid-log phase. The cells were harvested by centrifuging for 10 minutes at 5000 rpm in a Heraeus table top centrifuge and washed three times with 7H9 broth. The cell pellet was resuspended in plain 7H9 broth to get an A_600_ ~1. 100 μl of appropriate dilutions of this culture were plated on 7H11 plates supplemented with different concentrations of IPTG. The plates were incubated for 48–72 hours at 37°C. The absence of growth on plates without IPTG suggested essentiality of these genes. This experiment also enabled determination of the minimum IPTG concentration needed to support the growth of each conditional expression strain. KD strains were grown at this concentration of IPTG (referred to as minimum inducer required) specific for each strain in subsequent experiments unless otherwise stated.

### Growth kinetics of *inhA*, *rpoB* and *ftsZ* KD strains

The *inhA*, *rpoB* and *ftsZ* KD strains were grown in 7H9 broth supplemented with the pre-determined minimum IPTG concentration till they reached mid logarithmic phase. The cells were harvested by centrifuging for 10 minutes at 5000 rpm in a Heraeus table top centrifuge and washed three times with 7H9 broth. The cell pellet was resuspended in 7H9 broth and used as a starter culture for growth kinetic experiments. A master culture for each KD strain was prepared by diluting the starter culture to about 10^6^ CFU/ml. The culture was split into multiple aliquots and each culture aliquot was supplemented with a different concentration of IPTG. The growth was monitored by measuring A_600_ and enumerating the CFU over a period of 48–72 hours. Cultures sampled at the 30^th^ hour were used for Western blot analysis.

### Western blot analysis

10 ml cultures of wild-type *M*. *smegmatis* mc^2^155 and the *inhA*/KD/DO/*hemH*, *rpoB*/KD/DO and *ftsZ/*KD/DO strains were harvested at 30^th^ hour during the growth kinetic studies. The cell pellets were washed twice in 1X PBS and re-suspended in about 250–500 μl of 1X PBS containing a cocktail of protease inhibitors. The resulting cell suspension was transferred to a 2 ml screw cap tube containing about 0.1 g of 0.1 mm Zirconia beads. The cells were lysed by bead beating the cell suspension twice at 4500 rpm for 20 seconds each in a Mini-bead-beater. The clarified lysate obtained after centrifugation at 10,000 rpm for 5 minutes was used for protein estimation using Bradford reagent. 2 μg of total protein from each sample was resolved on SDS-PAGE and the proteins were blotted onto a Hybond nitrocellulose membrane (Amersham Hybond-ECL). The blots were probed with a 1:200,000 diluted polyclonal antisera raised in rabbits against either InhA or RpoB or FtsZ or SigA protein. This was followed by the treatment of the blots with secondary antisera conjugated to horse radish peroxidase. The blots were developed using an advanced ECL chemiluminescence substrate following manufacturer’s instruction.

### Microscopy

A culture inoculum having 10^5^ CFU/ml of *ftsZ*/KD/DO strain was prepared in 7H9 broth as described earlier. Culture was dispensed into a microtitre plate containing different concentrations of IPTG and incubated at 37°C. Following 48 hours of incubation, 10 μl aliquots of the culture were smeared onto a glass slide and heat fixed. The slides were stained using Carbol Fuschin (SIGMA) as described earlier [28]. The stained slides were washed thoroughly with water, air dried and observed under a light microscope (Zeiss-Axiolab) at 100X magnification.

### Minimum inhibitory concentration (MIC)

MIC of isoniazid (INH) and rifampicin (RIF) for wild-type *M*. *smegmatis* mc^2^155, *rpoB*/KD/DO, *inhA*/KD/DO/*hemH*, *InhA*/OE (generated by electroporating the recombinant plasmid pAZI9476 which had the *M*. *smegmatis inhA* gene cloned in the pMV261 vector [27] at BamHI and HindIII sites) were determined using the standard turbidometric method as reported earlier [29]. Briefly, an inoculum of culture having 10^5^ CFU/ml was incubated with different concentrations of IPTG (along the rows) and different concentrations of the antibiotic (along the columns). The plates were gently mixed and incubated at 37°C. The turbidity of each well was measured at 600 nm following 72 hours of incubation. The percent growth inhibition was calculated with respect to the growth observed at the optimal IPTG concentration for each KD strain without the antibiotic supplementation. Similarly, MIC of these compounds against the wild-type strain was determined using a culture of *M*. *smegmatis* mc^2^155. MIC assays were run with *M*. *tuberculosis* H37Rv strain using the standard RBMA method [30].

### Phenotypic screening

The screening conditions for *inhA*/KD/DO/*hemH* and *rpoB*/KD/DO strains were optimized by testing various parameters like IPTG concentration, method of end point reading and length of incubation at 37°C. A master culture with 10^5^ CFU/ml of each of the culture was prepared in 7H9 broth containing 18 μM IPTG for *inhA*/KD/DO/*hemH* strain and 200 μM IPTG for *rpoB*/KD/DO strain. Greiner 384-well plates were used for screening where in 40 μl of this master culture was dispensed per well containing either 1 μl of compound (test wells) or 1 μl of DMSO (control wells). Isoniazid and rifampicin were used as internal controls in each plate. Media with DMSO was used as media control and culture with DMSO as culture control. In parallel, test compounds were also screened against *M*. *smegmatis* mc^2^155. The plates were incubated at 37°C for 48 hours and A_600_ measured in a Spectramax plate reader. In each case, percent inhibition of growth in test wells was calculated with respect to the growth in the uninhibited control wells. The minimum inhibitory concentration (MIC) was defined as the lowest concentration of compound resulting in ≥ 80% growth inhibition following 48 hours of incubation at 37°C.

### Enzyme assays

InhA and RNA polymerase assays were performed to determine the 50% inhibitory concentrations (IC_50_) as described in Bhat *et al*., and Shirude *et al*. respectively [31, 32].

## Results

### Generation of IPTG inducible conditional expression vectors with single and double *lac* operators

**Fig 1:**
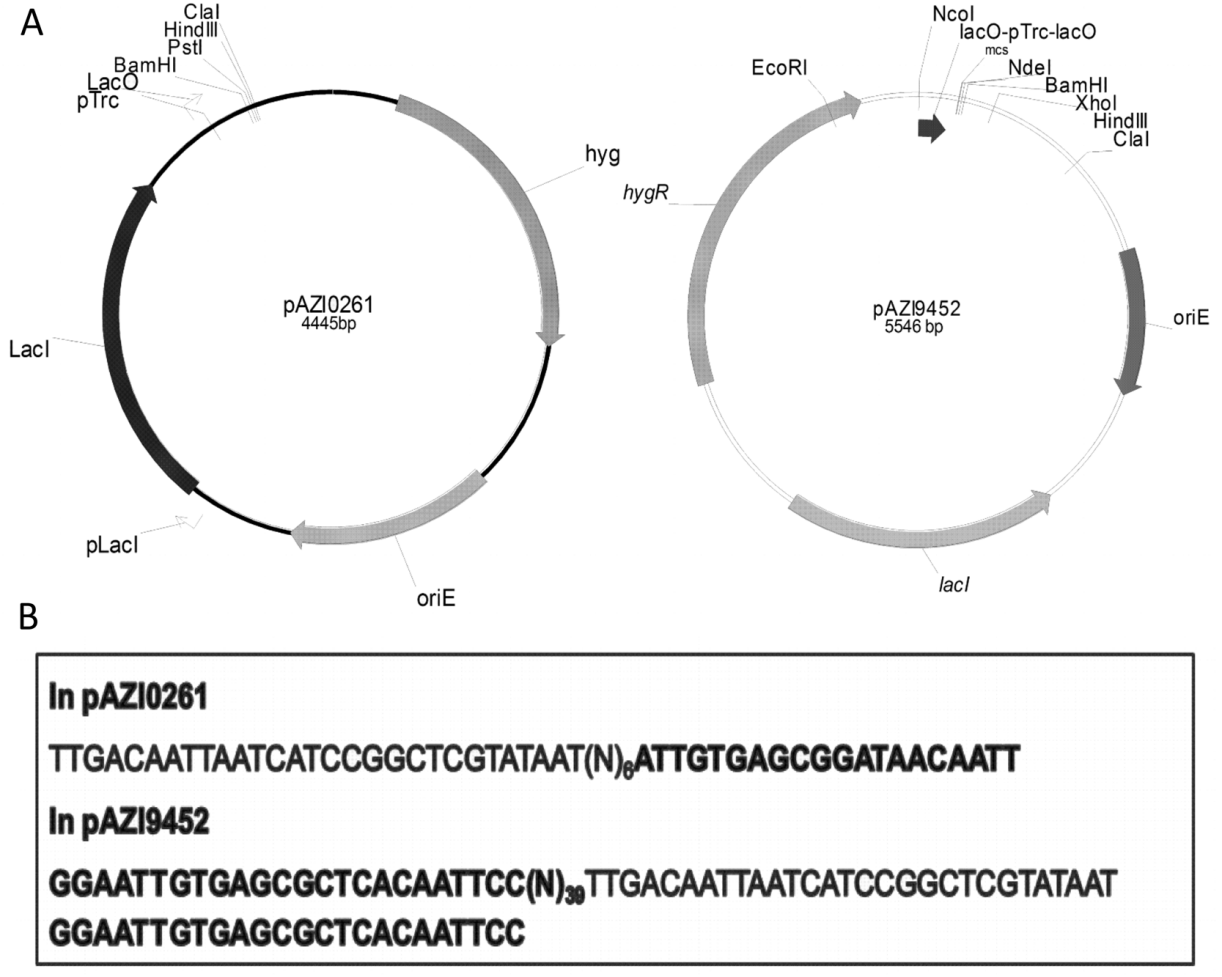
IPTG inducible conditional expression vectors with promoter-operator sequences. **(A)** Vector maps of conditional expression vectors with single lac operator (left) and double lac operator (right); **(B)** Promoter-operator sequences present in the two conditional expression vectors.

pAZI9018b is a replicating *E*. *coli*—mycobacterial shuttle vector with Ptrc promoter, whereas pT73.3 is an *E*. *coli* expression vector with T7 promoter. Two non-replicating conditional expression vectors pAZI0261 and pAZI9452 (Fig 1A) were constructed as described in materials and methods using pAZI9018b and pT73.3 as parent vectors respectively. Both the resultant plasmids possess an IPTG inducible promoter system, the former one with single *lac* operator and the later one with two *lac* operators as shown in Fig 1B.

### Analysis of β-galactosidase expression

**Fig 2:**
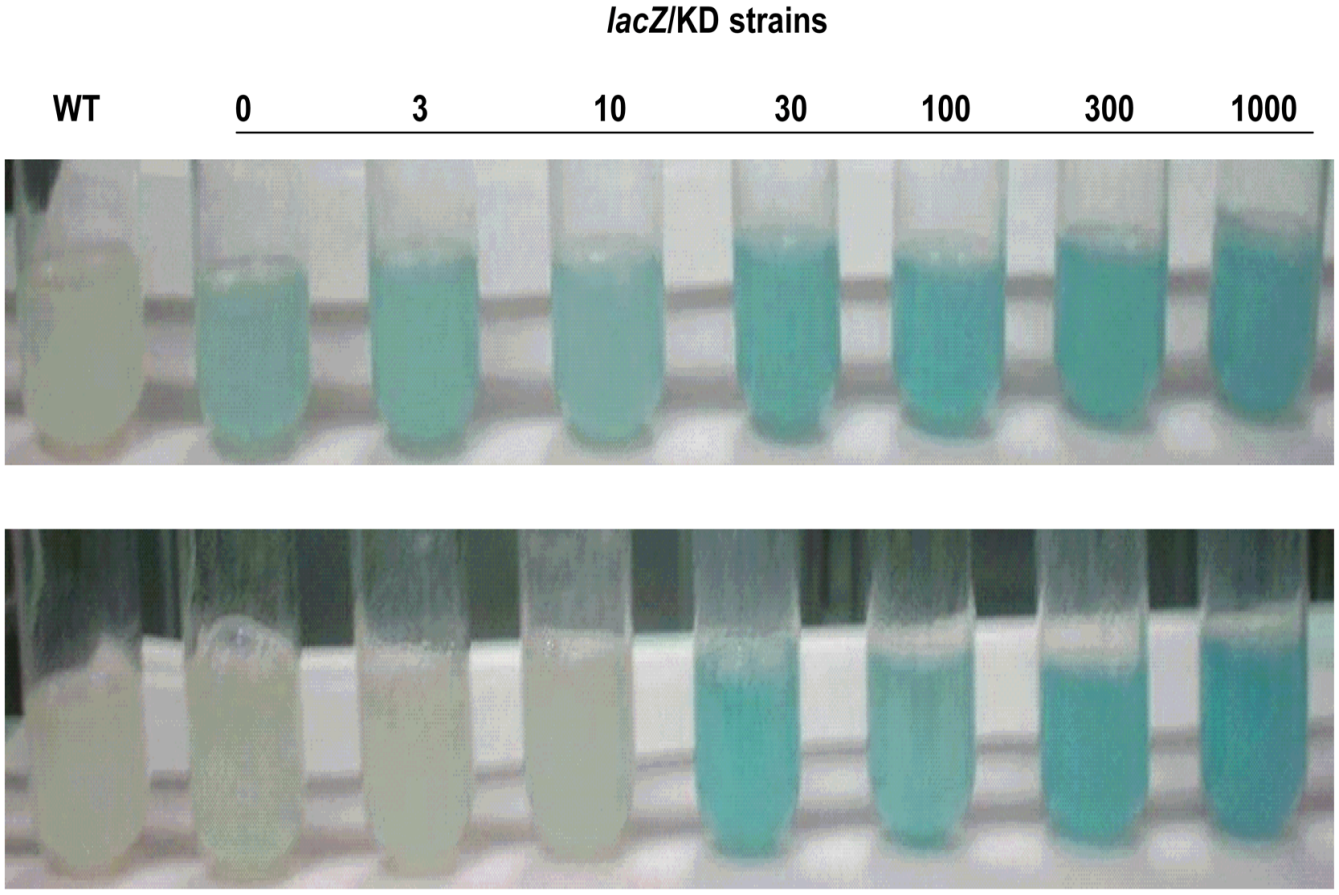
Assessment of regulation of β-galactosidase expression from the conditional expression vectors. *lacZ*/KD/SO (top panel) and *lacZ*/KD/DO (bottom panel) were grown at 37°C in the absence and the presence of different concentrations of IPTG and 40 μg/ml of X-gal. Wild-type *M*. *smegmatis* mc^2^155 (WT) was used as control which received 1000 μM IPTG and 40 μg/ml X-gal. The numbers indicate the μM IPTG concentrations used in the respective tubes.

In order to evaluate the ability of these two recombinant vectors, pAZI0261 and pAZI9452, to regulate gene expression as a function of added inducer, plasmids harbouring a *lacZ* gene were designed. We hypothesized that a dose-dependent and reliable expression is likely to be achieved with strains having a single copy of the gene of interest driven by a desirable promoter. To be able to achieve this, it was necessary to integrate the plasmids with *lacZ* into the chromosome using the attP-*int* sequence as mycobacteria lack a *lacZ* gene. In the case of pAZI9452, the *lacZ* gene was cloned to generate pBAN0195, while in the case of pAZI0261 the parent vector pAZI9018b was modified to retain the *lacZ* gene. The *attP-int* gene sequence was cloned into the recombinant plasmids (pBAN0195 and pAZI9018b with origin of mycobacterial replication removed) to generate pBAN0196 and pAZI0233, respectively (S1 Fig). Transformation of *M*. *smegmatis* with these non-replicating plasmids resulted in the generation of two recombinant strains, each with a single copy of *lacZ* gene under the control of Ptrc promoter. While the strain *lacZ*/KD/SO has a single *lac* operator, the strain *lacZ*/KD/DO has a double *lac* operator. A qualitative assessment of the β-galactosidase expression from *lacZ*/KD/SO and *lacZ*/KD/DO cultures grown with different concentrations of IPTG was performed using the chromogenic substrate X-gal. A blue colour developing due to the formation of 5,5’-dibromo-4,4’-dichloro-indigo from the hydrolyzed product 5-bromo-4-chloro-indoxyl of X-gal in these cultures would indicate the presence of β-galactosidase activity. The results shown in Fig 2 clearly suggested an IPTG dose-dependent expression of *lacZ* to be superior in the *lacZ*/KD/DO strain. A significant amount of leaky expression of β-galactosidase was seen in the *lacZ*/KD/SO strain even in the absence of IPTG, while no leaky expression was observed in the *lacZ*/KD/DO strain. A robust and reliable conditional expression vector is expected to provide a dose-dependent expression of cloned gene of interest with no leaky expression in the absence of an inducer. Thus, these results confirmed that the vector with two *lac* operators as a suitable conditional expression vector for the analysis of gene essentiality and vulnerability in mycobacteria.

### *rpoB* conditional expression validates the double operator IPTG inducible system

**Fig 3:**
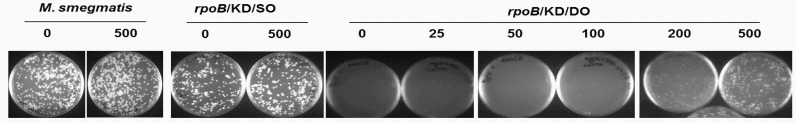
Minimum IPTG requirement of the *rpoB* conditional expression strains. Cultures of wild-type *M*. *smegmatis*, *rpoB*/KD/SO and *rpoB*/KD/DO were plated on 7H11 plates supplemented with 50 μg/ml hygromycin and different concentrations of IPTG. Wild-type *M*. *smegmatis* mc^2^155 served as control. The numbers above the agar plates indicate the μM IPTG concentration supplemented in the respective plates.

The DNA-dependent RNA polymerase is an essential multimeric enzyme in all bacteria that is required to maintain bacterial cell viability. The *rpoB* gene codes for the β subunit of RNA polymerase which is the molecular target of rifampicin, a front line drug in the treatment of tuberculosis. A conditional expression strain of the essential gene *rpoB* is not expected to grow in the absence of an inducer. In order to exert fine control over the level of gene expression, we created conditional expression strains of *M*. *smegmatis rpoB* as described in materials and methods, using both the single and double *lac* operator KD vectors. The genotype for each of the recombinant strain was confirmed using a set of PCR reactions with primers designed based on the genetic organization at the locus before and after a single cross over recombination event (S2 Fig). A representative agarose gel picture of the PCR screening results of recombinant colonies of *rpoB*/KD/DO is shown in S2 Fig A similar strategy was followed to identify the right recombinant strain in the case of *rpoB*/KD/SO as well (data not shown). After confirming the genotype by PCR, the minimum inducer requirement for growth of these two strains (*rpoB*/KD/SO and *rpoB/*KD/DO) was established by plating the cultures on 7H11 plates supplemented with different concentrations of IPTG. Wild-type *M*. *smegmatis* mc^2^155 plated on 0 and 500 μM IPTG containing plates showed no difference in growth (Fig 3) and served as a comparator for the colony size and morphology. While no growth could be detected in the absence of IPTG with the *rpoB*/KD/DO strain having a double *lac* operator system, colonies similar to wild-type *M*. *smegmatis* grew only on plates supplemented with 500 μM IPTG (Fig 3). Strikingly, the *rpoB*/KD/SO strain with a single *lac* operator grew as well as the wild-type mc^2^155 strain even in the absence of IPTG, thereby highlighting the presence of a significant level of leaky expression (Fig 3). The results obtained from this experiment further confirmed the suitability of the conditional expression vector with two *lac* operators for the evaluation of gene essentiality. Further evaluation of this system was performed by analysing the growth kinetics of conditional expression strains of *rpoB* as well as other known essential genes, *inhA* and *ftsZ*.

### Growth kinetic profiles of *rpoB*, *inhA and ftsZ* conditional expression strains

**Fig 4:**
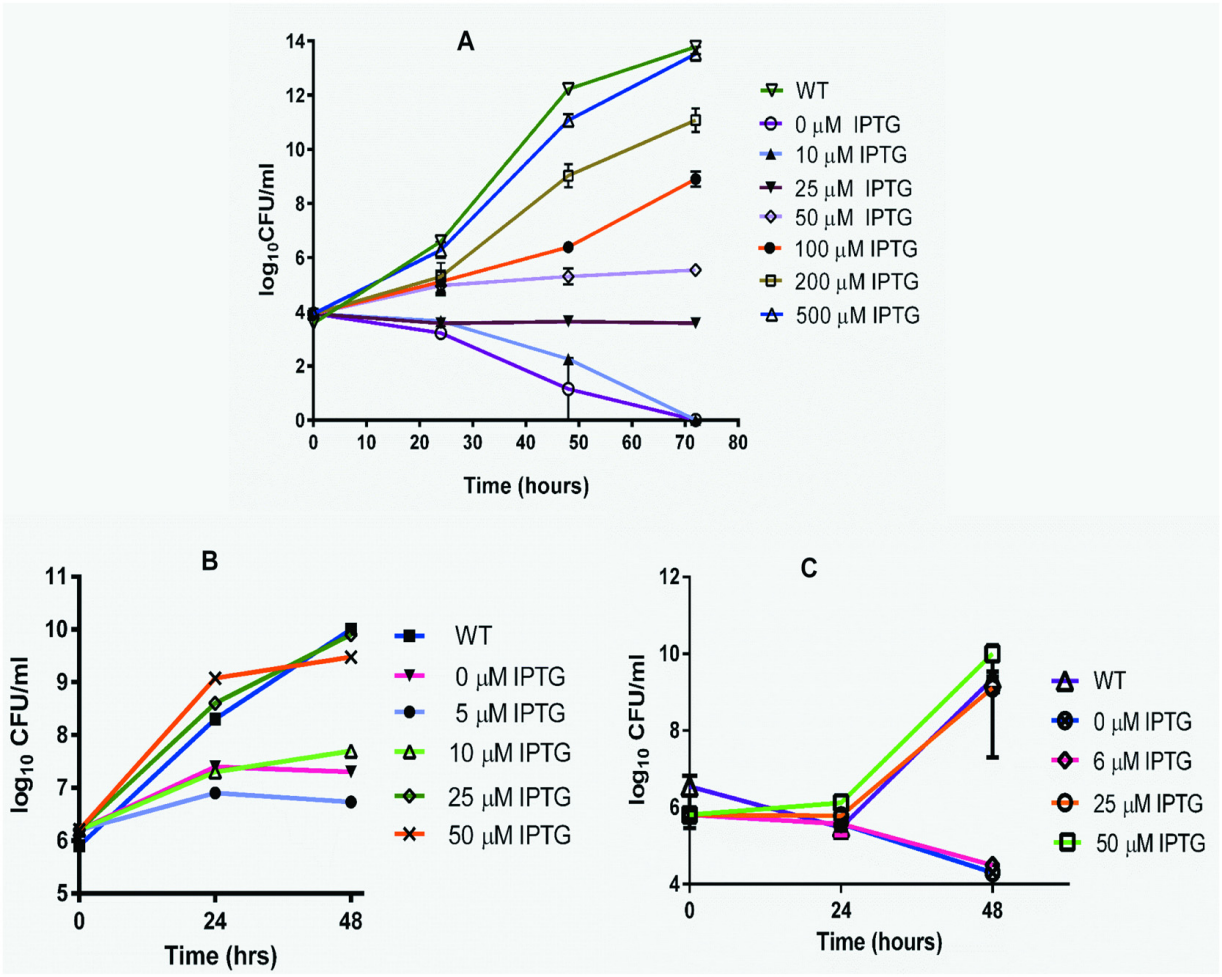
Growth kinetics of *rpoB*, *inhA* and *ftsZ* KD strains. KD cultures grown with minimum IPTG required were harvested, washed and used for preparing inoculum of cultures for growth kinetic studies. A master culture of each KD strain with ~10^6^ CFU/ml was prepared, split into many aliquots where each aliquot received a different IPTG concentration. They were grown at 37°C. The effect on the growth was monitored by measuring the survivors periodically by plating them on 7H11 plates supplemented with IPTG. *rpoB* (**A**), *inhA* (**B**) and *ftsZ* (**C**). The graphs are representative results obtained from 3 independent experiments.

A conditional expression strain generated through single cross over recombination results in an altered genotype in the cell because of the insertion of the entire plasmid at the genomic loci. Hence, unambiguous conclusions can be drawn about the phenotypic changes of a conditional expression strain if the gene of interest is located as a single gene or is the last gene in its transcriptional unit. However, if the gene of interest is either in the beginning or in the middle of an operon, conditional expression strains of such genes will need to be complemented with the gene/s present downstream to overcome potential polar effects. Towards this, we examined the genomic organization and transcriptional units of *M*. *smegmatis inhA*, *rpoB* and *ftsZ* genes. Among these, *rpoB* and *ftsZ* were found to be single genes in their respective transcription units. However, *inhA* was found to be in an operon with *fabG* on its 5’ end and *hemH* on its 3’ end, very similar to the genomic organisation that is found in *M*. *tuberculosis* [33]. HemH, a ferrochelatase involved in the heme biosynthetic pathway, is an ortholog of the essential gene *hemZ* of *M*. *tuberculosis* [34] with about 70% sequence identity at the protein level (data not shown). If *hemH* is essential in *M*. *smegmatis* as well, it would be hard to decipher if the phenotypic changes observed with *inhA*/KD/DO strain is due to the modulation in expression of *inhA* gene alone or *hemH* gene alone or a combined effect of down regulating both these genes. In order to overcome this challenge, a *hemH* full length gene was cloned downstream of the hsp60 promoter in pAZI272 vector to achieve constitutive expression. This plasmid was electroporated into *inhA*/KD/DO strain to generate a recombinant strain designate as *inhA*/KD/DO/*hemH*. Conditional expression strain of *rpoB* and *ftsZ* was generated as described in the materials and methods. All the three strains were used for generating growth kinetic profiles in the presence and the absence of IPTG. The minimum inducer dependency for growth of these strains determined on 7H11 plates supplemented with different IPTG concentrations established that the *rpoB* KD strain needed 500 μM of IPTG, while the *ftsZ* and *inhA* KD strains required 25 μM of IPTG each to support growth. These KD strains were then grown in 7H9 broth supplemented with the specified IPTG concentrations (500 μM for *rpoB* and 25 μM for *inhA* and *ftsZ*) until the cultures reached an optical density (A_600_) of about 0.2. The cells were harvested, washed, resuspended in 7H9 broth to be used as inoculum for the growth kinetic studies. Master cultures with ~10^6^ CFU/ml of each of these strains were prepared in 7H9 broth supplemented with 50 μg/ml hygromycin and then split into many aliquots, where each aliquot was supplemented with a different IPTG concentration including a control culture with no IPTG (to assess the inducer withdrawal effect). These cultures were grown at 37°C in a shaker incubator at ~150 rpm and the growth was monitored by measuring absorbance as well as colony forming units (CFU) periodically by spreading appropriate dilutions of the culture on 7H11 plates supplemented with IPTG (at minimum concentration required) to enumerate the number of bacterial survivors. An aliquot of the same culture was also plated on 7H11 plates without IPTG to ensure that revertants did not arise during the course of this experiment. The growth kinetic graphs were drawn by plotting colony forming units against time for each KD strain. Fig 4 shows the growth kinetics profiles of *rpoB*, *inhA* and *ftsZ* KD strains. While suboptimal concentrations of IPTG lead to bactericidal effect in the case of *rpoB* and *ftsZ* KD strains, a bacteriostatic effect was seen in the case of an *inhA* KD strain. These results confirmed the essentiality of *rpoB*, *inhA* and *ftsZ* genes for the survival of *M*. *smegmatis* and highlighted the likely vulnerability of these targets if inhibited by specific small molecule inhibitors. Furthermore, the bactericidal/bacteriostatic effect seen could be attributed to the reduction in the expression levels of target protein as the phenotype is specifically observed only in cultures with suboptimal IPTG concentrations and not with either the wild-type mc^2^155 strain or the knockdown strain grown with an optimal concentration of the inducer.

**Fig 5:**
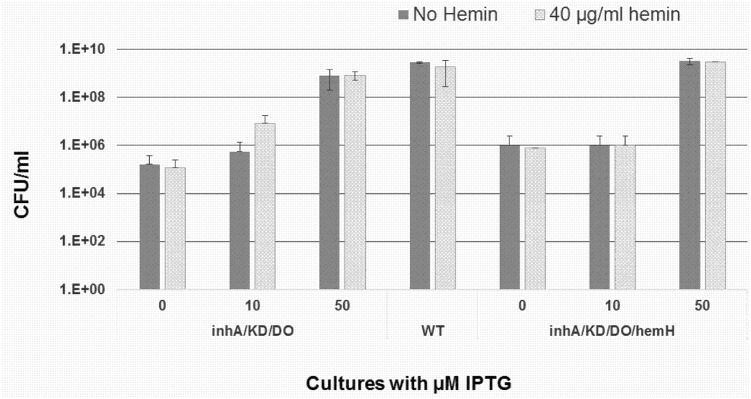
Growth dependence of *inhA*/KD/DO on hemin. Wild type *M*. *smegmatis* (WT), *inhA*/KD/DO and *inhA*/KD/DO/*hemH* were grown till mid log phase, washed and dilutions plated on two sets of 7H11 plates, one set supplemented with 50 μg/ml hygromycin, 40 μg/ml hemin and either 0, 10 or 50 μM IPTG, another set supplemented with 50 μg/ml hygromycin and either 0, 10 or 50 μM IPTG. Solid bars (no hemin (0H), shaded bars (40 μg/ml hemin (40H)). This data is representative of 2 independent experiments.

The *inhA*/KD/DO/*hemH* strain grew as well as the wild-type mc^2^155 strain only in the presence of 25–50 μM of IPTG. It is likely that at this inducer concentration, enough quantities of both *inhA* and *hemH* gene products are produced within the cell. However, at a suboptimal IPTG concentration, for example 10 μM IPTG, both of these proteins might not be produced in sufficient quantities to support optimal growth. As per the observations made by Parish *et al*., *hemZ* knockouts are auxotrophic for hemin [34]. If *inhA*/KD/DO culture grown in the presence of 10 μM IPTG is supplemented with hemin, one would expect the strain to grow better than if grown only in the presence of IPTG as this would ensure sufficient levels of both InhA and heme are synthesized. Based on this hypothesis, both the *inhA*/KD/DO/*hemH* and *inhA*/KD/DO strains were plated on 7H11 plates supplemented with 0, 10 and 50 μM IPTG concentrations, both in the absence and presence of 40 μg/ml of hemin. As seen in Fig 5, hemin supplementation of the *inhA*/KD/DO culture at 10 μM IPTG could improve the growth only slightly (by ten-fold), while no difference could be observed between the cultures with and without hemin either at 0 μM IPTG (no expression) or 50 μM IPTG (maximal expression). Similarly, the presence or absence of hemin in the *inhA*/KD/DO/*hemH* culture at any of the IPTG concentrations did not make any significant difference to the growth as the complemented *hemH* gene is functional and supporting the growth of this strain. This experiment provided compelling evidence for the expression of ferrochelatase encoded by the *hemH* gene in the complemented strain of *inhA* KD and clearly attributes the observed phenotype of this strain to be solely due to the reduction in the InhA protein levels.

To corroborate that the observed bactericidal or the bacteriostatic effect is indeed due to the reduction of specific proteins, namely, InhA, RpoB or FtsZ, knockdown cultures of each of these genes were sampled at specific time points during the growth kinetics study to assess the intracellular levels of InhA, RpoB and FtsZ proteins.

### Western blot corroborates growth kinetic phenotype

**Fig 6:**
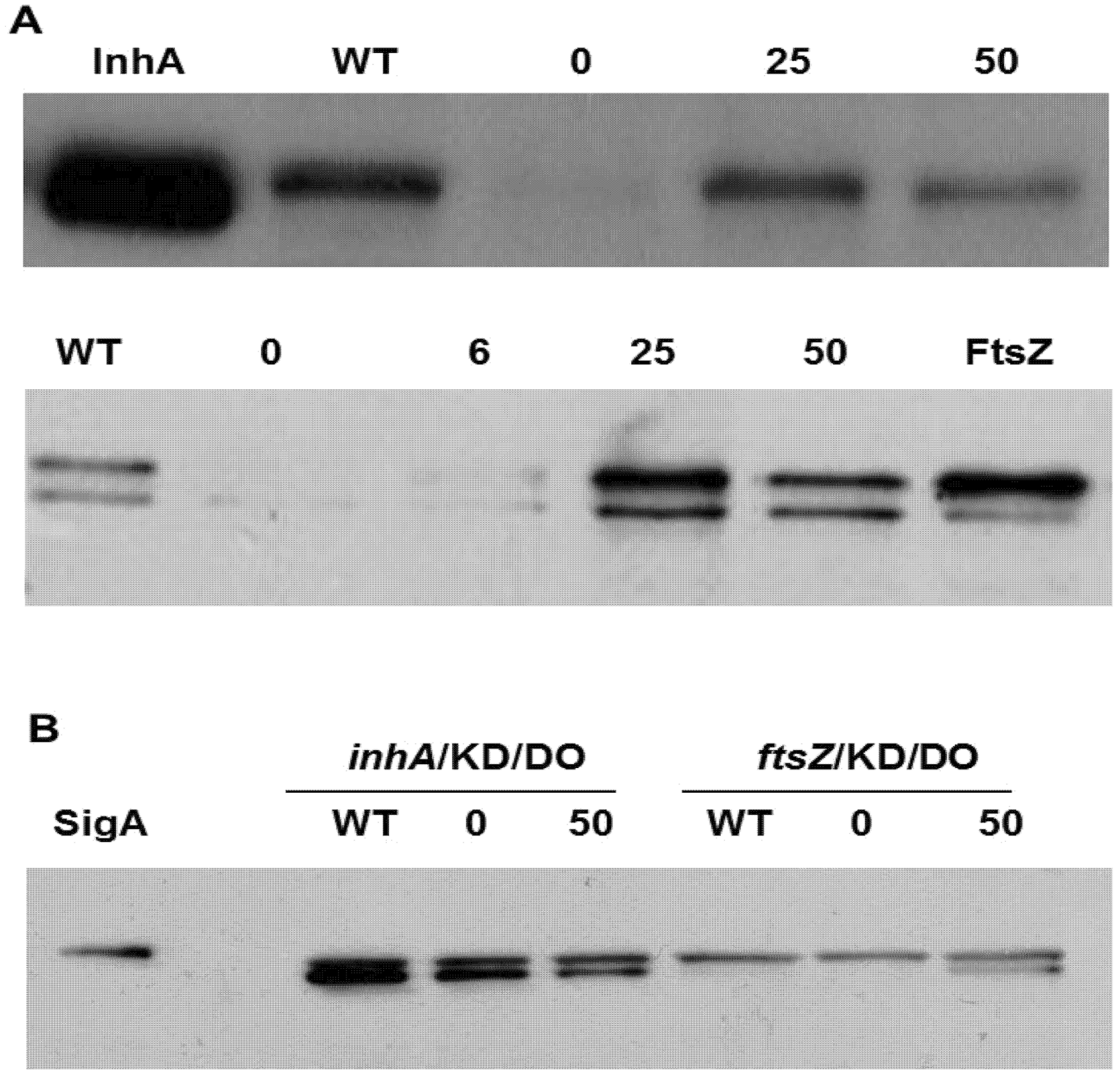
Intracellular levels of InhA, FtsZ and SigA proteins. Cultures of *inhA*/KD/DO/*hemH* and *ftsZ*/KD/DO strains were sampled at 30 hours from the start of growth kinetic studies (Fig 4). The harvested cells were lysed by bead beating in 1X PBS. About 2 μg protein content of each of the clarified lysate was loaded per lane (except in *ftsZ*/KD/DO for SigA where 1 μg total protein per well was used). The proteins separated on SDS-PAGE were blotted on to nitrocellulose membrane and probed with 1:200,000 diluted antisera of InhA, FtsZ (**A**) and SigA (**B**). Purified proteins of InhA, FtsZ and SigA were used as molecular size reference. *M*. *smegmatis* (WT) treated in a similar way was used as control.

Two micrograms of total proteins from the culture lysates of *inhA*/KD/DO/*hemH*, *rpoB*/KD/DO and *ftsZ/*KD/DO strains were resolved on SDS-PAGE, blotted onto nitrocellulose membranes. The blots were probed with polyclonal antisera raised in rabbits against either InhA or RpoB or FtsZ protein. The results from the Western blot analysis (Fig 6A) indicated that the *inhA*/KD/DO/*hemH* and *ftsZ/*KD/DO strains grown with optimal IPTG concentration had intracellular levels of InhA and FtsZ proteins comparable to those present in the wild-type strain. While it was same or higher in case of FtsZ, it was slightly lower than the wild-type levels for InhA. The small differences observed in the expression levels InhA and FtsZ could be due to the heterologous promoter (Ptrc) driving the expression of these essential genes in these conditional expression strains. When they were grown in the presence of suboptimal concentrations of IPTG, the levels of these proteins were significantly lower in the KD strains in comparison to the levels observed in the wild-type *M*. *smegmatis* mc^2^155 cells. A clear difference in the protein levels could not be inferred in the case of the *rpoB* KD strain, because of the high background seen in the autoradiogram. This could probably be due to the high level of non-specific reactions from the polyclonal antibody used for probing (data not shown).

Withdrawal of inducer from a culture of a knockdown strain is expected to result in the reduction of specific protein levels whose expression is driven by the inducible promoter and not affect the production of other cellular proteins. By monitoring the intracellular levels of a house keeping protein like Sigma factor 70 (SigA) in the culture of a knockdown strain grown under inducer depleted conditions, it is possible to generate experimental evidence on the specificity of the observed knock down effect. An aliquot of culture lysates of *inhA* and *ftsZ* KD strains which were used for Western blot analysis with InhA and FtsZ antisera were also probed with SigA antisera. This Western blot showed SigA levels in the *inhA*/KD/DO/*hemH* and *ftsZ/*KD/DO strains grown without IPTG to be similar to the levels observed in the same strains grown with optimal IPTG concentration and also the wild-type *M*. *smegmatis* strain (Fig 6B). Thus, the Western blot data enabled linking the altered growth phenotype observed with *inhA* and *ftsZ* conditional expression strains upon inducer withdrawal to the specific reduction in the intracellular levels of the respective proteins.

### Confirmation of conditional expression through filamentation and MIC modulation studies

**Fig 7:**
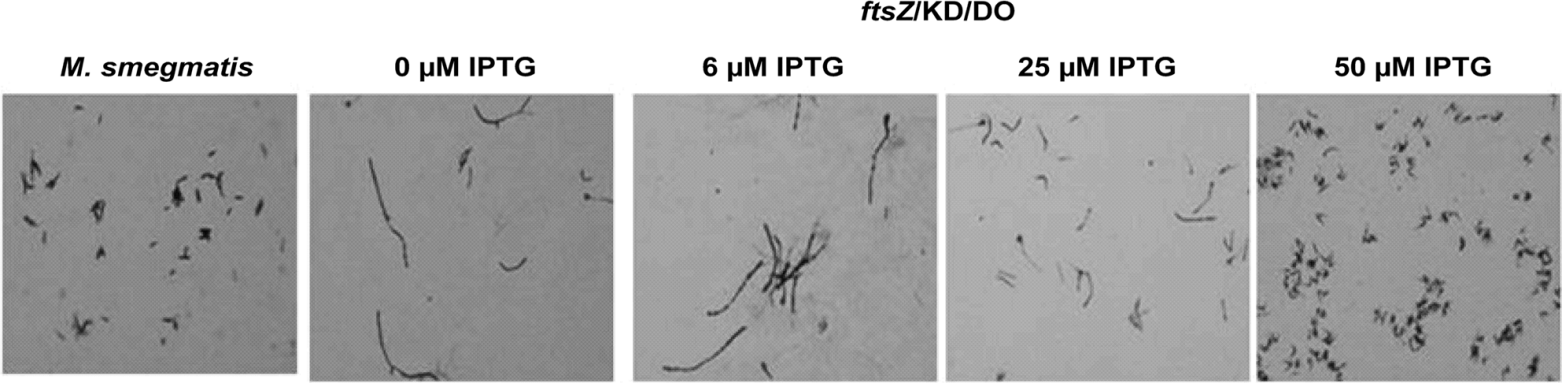
Filamentation assay. *M*. *smegmatis* and *ftsZ*/KD/DO cultures grown at 37°C with different concentrations of IPTG were sampled at 48 hours of incubation. A smear of these cells on the microscope slides were stained with Carbol Fuschin followed by light microscopy.

FtsZ is an essential protein in the cell division process. A reduction in the intracellular levels of this protein *via* genetic knockdown or chemical inhibition is known to result in an extensive filamentation of the bacterial cells [35]. Dziadek *et al* have also demonstrated that the cells undergo filamentation and lyse subsequently following depletion of the intracellular levels of FtsZ protein. This distinctive phenotypic property of FtsZ has been exploited in the evaluation of several mycobacterial conditional expression vectors developed till date [5, 14]. On similar lines, we stained the *ftsZ* conditional expression strain grown with and without IPTG and observed for morphological changes using light microscopy. Filamentation of the bacterial cells were observed only when the *ftsZ*/KD/DO strain was grown with suboptimal IPTG concentration and not when grown with optimal IPTG concentration as shown in Fig 7. The wild-type mc^2^155 strain grown in the presence or the absence of IPTG did not show any such phenotypic change (data not shown). This result corroborated that the bactericidal effect and the filamentation observed with the *ftsZ*/KD/DO strain when grown without inducer is indeed due to the depletion of intracellular FtsZ levels as supported by the Western blot analysis.

InhA and RpoB proteins are the molecular targets of the front line TB drugs, isoniazid and rifampicin, respectively [36, 37]. The reduction in intracellular levels of these target proteins is likely to reflect in the reduction of their respective MIC as well. Due to the lack of a bonafide FtsZ inhibitor in our collection, all MIC assays were performed only with the *inhA* and *rpoB* conditional expression strains using a standard turbidometric method. The culture inoculum for this study was prepared as described in materials and methods. The wild-type *M*. *smegmatis* mc^2^155 strain was used as a control. The MIC data presented in Tables 2 and 3 demonstrate the modulation in MIC of isoniazid for *inhA*/KD/DO strain (~8-fold) and that of rifampicin for *rpoB*/KD/DO strain (4 to 16-fold) respectively when the inducer concentration is reduced. However, the MIC of rifampicin for *inhA* KD strain and the MIC of isoniazid for *rpoB* KD strain remained the same as that for the wild-type *M*. *smegmatis* strain. The results confirmed that the modulation in MIC of rifampicin and isoniazid are due to the reduction in the respective target protein levels. Additionally, this data also provided an indication that these strains could be used to explore the possibility of identifying new inhibitors for these clinically validated targets in a cell based screening program.

**Table 2:** MIC modulation.

IPTG (μM)	MIC (μg/ml) of Isoniazid (INH) in	
	*inhA*/KD/DO/*hemH*	*rpoB*/KD/DO
12.5	0.125	*
25	1	*
50	1	*
200	2	2

*No growth detected

MIC of INH for *M*. *smegmatis* mc^2^155 = 2 μg/ml.

**Table 3:** MIC modulation.

IPTG (μM)	MIC (μg/ml) of Rifampicin (RIF) in	
	*inhA*/KD/DO/*hemH*	*rpoB*/KD/DO
100	8	0.5
200	8	2
400	8	4
800	8	4

MIC of RIF for *M*. *smegmatis* mc^2^155 = 8 μg/ml.

### Identification of novel inhibitors against InhA and RNA polymerase

**Fig 8:**
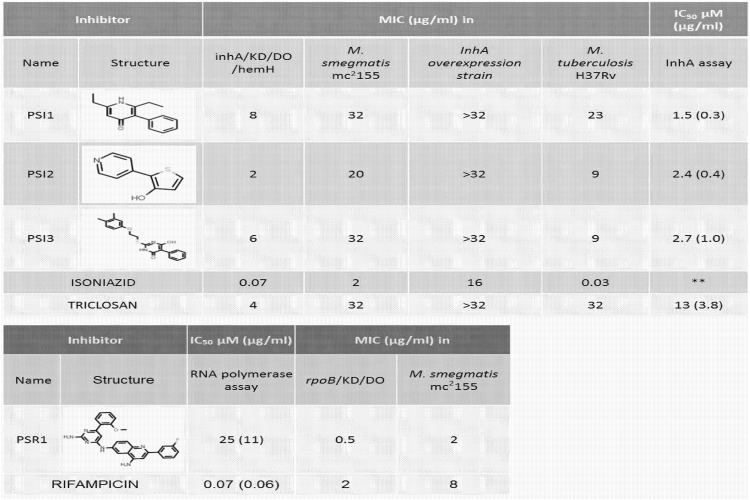
Hits from phenotypic screen.

The details of the assay conditions used for the inhibitor screening are described in materials and methods. A focused set of ~1200 small molecules from the AstraZeneca corporate collection with MIC against *M*. *smegmatis* mc^2^155 (MIC ≤64 μg/ml) was chosen for this screen. As three strains (wild-type *M*. *smegmatis*, *inhA*/KD/DO/*hemH*, *rpoB*/KD/DO) had to be used in parallel to perform the screen, we chose to perform the initial screen using a 5-point compound concentration ranging from 2 to 32 μM with a two-fold change in compound concentration in the adjacent wells. The minimum concentration of compound that produced an 80% growth inhibition in comparison to the uninhibited control (no compound) was taken as the MIC for each strain. Any compound that showed a minimum of four-fold down-shift in MIC in the conditional expression strain as compared to the MIC in the wild-type strain was tested again in a 10-point (concentration ranging from 0.2 to 100 μM) MIC assay. Compounds which showed a reproducible four-fold or more reduction in the 10-point MIC assay were tested in InhA and RNA polymerase cell free enzyme assays, respectively. Isoniazid, triclosan and rifampicin were used as reference inhibitors in these screens. Fig 8 shows a list of novel compounds identified through this screen that demonstrate a four-fold or more shift in the MIC values with a concomitant IC_50_ ≤ 50 μM in the specific enzymatic assay. These hits were also found to inhibit the growth of wild-type *M*. *tuberculosis* H37Rv strain (Fig 8). The mode of action of the identified InhA inhibitors was re-confirmed when they exhibited an up-shift in MIC in a *M*. *smegmatis* InhA overexpression strain as compared to the MIC observed in a wild-type strain (Fig 8).

## Discussion

The high attrition rate encountered during the drug discovery and development process has mandated the importance of choosing high value targets to increase the chances of success in identifying new drugs [38]. Target identification and validation thus becomes a critical first step in a drug discovery program to prove the essentiality of a chosen target in order to establish a link with the disease biology [39, 40]. While the target identification mainly involves literature and bioinformatics analysis, the process of target validation involves deleting the gene of interest from the chosen pathogen. While the gene deletion strategy provides gene essentiality information under the standard laboratory growth conditions many bacteria, especially pathogenic bacteria including mycobacteria which are known to survive under different physiological environments making it important to understand the essentiality of chosen targets under these altered physiological conditions as well [41]. Conditional expression strains provide an alternative means to study the requirement of a gene for cell survival under a variety of growth conditions including the survival in various animal models. The ability to readily modulate gene expression when required with addition or withdrawal of an inducer has enabled the understanding of vulnerability of a plethora of target genes under *in vitro* and *in vivo* growth conditions [10, 42]. In bacteria, conditional expression strains have been generated either by expressing antisense message or by swapping the native promoter of the gene of interest with an inducible promoter through the generation of a single cross over recombinant or by providing a complementing copy of the gene of interest under an inducible promoter in a knockout strain [43]. Each of these approaches has certain advantages and some limitations. In the case of antisense expression strategy, even a small amount of leaky expression will make it difficult to isolate the required recombinant strain. Additionally, it would be hard to establish off-target effects. Although, the knockout strategy with an inducible complementing copy of the gene of interest is ideal, it is often time and labour intensive in mycobacteria because of the long doubling time and the requirement to work under biosafety level 3 conditions. In the case of a single cross over recombinant generated for genes in an operon, the potential downstream polar effects may interfere with the overall interpretation of the phenotypic changes observed. In order to circumvent these limitations, we chose to use a strategy of generating single cross over recombinants which is easier and faster and where necessary, complement the gene/s downstream under a constitutive promoter to overcome potential polar effects. An IPTG inducible promoter system (Ptrc promoter, *lac* operator and *lac* repressor) was used to develop a new conditional expression vector for mycobacteria, as it has been shown earlier that IPTG has no detrimental effect on the growth of mycobacteria even at high concentrations [11, 44]. Previous studies on the successful use of IPTG in animal models to induce gene expression [10, 45] also positively influenced our decision to work with an IPTG inducible system as one of the objectives of our work was to use this system in *M*. *tuberculosis* to assess gene essentiality both under *in vitro* and *in vivo* growth conditions.

Conditional expression vectors can be employed to study essentiality and vulnerability of genes only if they satisfy the following two key criteria (i) inducer dose-dependent regulation of gene expression and (ii) tight regulation of gene expression. While the former criterion is required to delineate vulnerability information (quantum of reduction in gene expression that results in bactericidal effect), the later one is required for generating gene essentiality information. There is a preference for highly vulnerable targets over other targets in drug discovery programs, as small reduction in the target protein levels affects the bacterial cell viability. Wei and co-workers [46] have demonstrated that conditional expression strains have the capability to distinguish vulnerable targets like *rpoB*, *inhA*, *gyrA* from those that are not vulnerable, like the *dfr* gene. However, inducer dose-dependent regulation in gene expression could be a fine interplay between the amounts of repressor, operator and inducer present in the cell and the affinity of the repressor to the inducer and operator. The ability of an inducible system to regulate expression as a function of inducer concentration has been assessed through the expression of reporter genes like *gfp* (green fluorescent protein) or *lacZ* [5–6, 11 and 46]. The qualitative analysis of *lacZ* expression performed in this study suggested that the system with double *lac* operator provided better regulation in expression as evidenced by the expression of β-galactosidase only in presence of IPTG. This was further substantiated by the IPTG dose-dependent growth kinetics observed with *rpoB* KD strain. However, the ‘all or none’ kind of growth kinetic profile observed for the other essential genes such as *inhA* and *ftsZ* in this study indicated that the conditional phenotype of a particular KD strain is also influenced by the gene that is being regulated.

To achieve a total knock down of gene expression as it happens in the case of gene knockouts, a conditional expression system is expected to have a very tight regulation to achieve ‘zero level’ of expression. Since the tightness of regulation is a function of repressor-operator interaction, some of the earlier studies have either increased the number of operator sequences around the promoter region to increase the cumulative affinity and / or overexpressed *lac* repressor [11] or used a combination of altered *lac* repressor and operators [47, 48]. We decided to evaluate a pair of palindromic *lac* operator sequences by placing one on either side of the Ptrc promoter in a mycobacterial non-replicating vector. The ability to repress the expression of *lacZ* reporter gene was assessed by employing a simple qualitative colorimetric assay. The absence of blue colour in *lacZ*/KD/DO strain grown without IPTG clearly confirmed that the palindromic *lac* operator sequence could provide sufficient repression to achieve ‘zero level’ of expression. A complete lack of growth of the *rpoB* KD strain in the absence of IPTG further confirmed the robustness of regulation. Our data also suggested that a single *lac* operator is not sufficient enough to achieve significant repression even when there is a single copy of the gene of interest.

Results obtained in this study with the double *lac* operator containing IPTG inducible system clearly suggests that it can also be employed for recombinant protein expression in *E*. *coli*. Similar to the T7 expression vectors, this vector is particularly useful for expression of toxic proteins whose expression prior to induction could prove detrimental to the host cell. However, unlike the T7 promoter containing expression vectors, the vector developed in this study doesn’t require any specific host strain for gene expression.

Although the reporter gene expression is indicative of the quality of the conditional expression vector, the true value of such vectors would be demonstrated by the phenotype of conditional expression strains of essential genes grown in the absence of an inducer. To further demonstrate the value of our approach, we evaluated the conditional expression of three essential genes in *M*. *smegmatis*, namely, *inhA*, *rpoB* and *ftsZ*. Two of the chosen genes, *inhA* and *rpoB* are the molecular targets of the front line TB drugs, isoniazid and rifampicin respectively and therefore are clinically validated. *ftsZ*, another essential gene has been used as a tool to study a number of conditional expression strains by virtue of its ability to induce filamentation when the functional enzyme levels are reduced in bacterial cells [49]. The choice of these genes for evaluation was also driven by the availability of tools like specific antibodies and antibiotics. The cell viability studies conducted with the three knockdown strains upon withdrawal of the inducer demonstrated that the vector with two *lac* operators provides essentiality information without any ambiguity. On the other hand, study of target vulnerability would be possible with genes which exhibit growth kinetic profile similar to the one obtained with *rpoB KD strain*. However, vulnerability assessment for *rpoB* could not be done in this study because of the inability to measure the relative intracellular levels of β-subunit of RNA polymerase between samples as a result of the poor specificity of the antibody against this protein.

It is necessary to demonstrate that the phenotypic changes observed in conditional expression strains under suboptimal growth conditions are due to the specific reduction in the protein levels being knocked down and that the other cellular protein levels are unaffected. Towards this, we have employed several tools such as Western blot analysis, MIC assay and filamentation assay with appropriate internal controls as has been done previously [6–18, 46]. We obtained unambiguous results by Western blot analysis for the *inhA* and *ftsZ* KD strains suggesting the phenotype observed is due to the specific reduction in InhA and FtsZ proteins respectively. SigA levels were same in all samples of *inhA* and *ftsZ* KD strains, irrespective of the inducer concentration and it was similar to the levels observed in the wild-type *M*. *smegmatis* strain. However, the levels of *inhA* and *ftsZ* proteins were several fold lower in their respective conditional expression strains when grown in the absence of IPTG compared to the levels observed in the wild-type strain.

An alteration in the intracellular target levels, is likely to induce growth defect and also exhibit altered phenotypic properties in a bacterial cell. We decided to employ the filamentation assay for characterising the *ftsZ* KD strain and MIC modulation assay for the *inhA* and *rpoB* KD strains to assess the additional phenotypic changes. At suboptimal IPTG concentrations, the *ftsZ*/KD strain clearly turned to a filamentous morphology which changed to normal growth morphology as IPTG concentrations reached optimal levels. The morphology of the wild-type *M*. *smegamtis* mc^2^155 did not exhibit any change with altered IPTG levels. Similarly, the MIC assay with *inhA* and *rpoB* KD strains helped in attributing the MIC modulation to reduced target protein levels. Isoniazid and Rifampicin are two potent inhibitors of *M*. *tuberculosis* with MIC in the range of 0.01 μg/ml. Although isoniazid is equally potent on both *M*. *smegmatis* and *M*. *tuberculosis*, rifampicin is poorly active against *M*. *smegmatis* (MIC = 8 μg/ml). Therefore, we hypothesized that the *rpoB*/KD strain should become hyper sensitive to rifampicin, if the RpoB levels were lowered by reducing the concentration of IPTG supplemented in the growth medium. On similar lines, *inhA*/KD strain will also become more sensitive to isoniazid at IPTG concentrations less than 50 μg/ml. To prove this hypothesis, the MIC of isoniazid and rifampicin for these two KD strains were determined at different IPTG concentrations using standard turbidometric method. As expected, a reduction in the MIC for rifampicin in the *rpoB*/KD/DO strain (about 16-fold) and the isoniazid MIC for the *inhA*/KD strain (about 8-fold) was observed. The MIC of rifampicin in the *inhA*/KD/DO strain and the MIC of isoniazid in the *rpoB*/KD/DO strain was found to be the same as the MIC observed for the wild-type *M*. *smegmatis* strain. This data also suggested the possibility of using these strains for a phenotypic screen to identify specific inhibitors. As reported earlier [50–52], by virtue of becoming hyper sensitive to specific target inhibitors as a result of lowered target levels, a conditional expression strain at a suboptimal inducer concentration has the ability to identify specific inhibitors from a screen which could be missed if screened against the wild-type strain. In order to find new drugs for the treatment of tuberculosis, phenotypic screens should be performed in *M*. *tuberculosis*, the causative agent of tuberculosis in humans. However, the long doubling time of *M*. *tuberculosis* and the requirement for stringent biosafety level 3 laboratory slows down the pace at which large compound libraries can be screened to identify novel growth inhibitors. In order to accelerate the screening process and to generate a proof of concept, we performed the phenotypic screen using *M*. *smegmatis* strain as a surrogate host and confirmed that the identified hits were equally active against *M*. *tuberculosis*. The attractive hits identified in this screen can be further optimized through a systematic medicinal chemistry approach by tracking the MIC of compounds against *M*. *tuberculosis*.

The present study, by employing a variety of genes such as *inhA*, *rpoB* and *ftsZ* has validated the IPTG inducible system with double *lac* operator to be suitable for establishing the essentiality and vulnerability in *M*. *smegmatis*. Similar validation studies are required using *M*. *tuberculosis* strain to expand the use of this vector for establishing gene essentiality and vulnerability of target genes before selecting them for a drug discovery program.

## Conclusion

IPTG inducible expression systems are routinely used in *E*. *coli* for expression of homologous and heterologous proteins. Reports regarding the use of this system in mycobacteria are limited. This study establishes the utility of the *E*. *coli* IPTG inducible system for conditional expression of target genes in the fast growing *M*. *smegmatis*. By using two palindromic *lac* operators, we demonstrate that the regulation of gene expression is more robust and tightly controlled. Additionally, we extend the utility of these conditional expression strains for the identification of target specific inhibitors.

## Supporting Information

S1 Fig Conditional expression plasmids with *lacZ*. Plasmids with *lacZ* and *attP-int* sequences used for the evaluation of regulation of expression from the IPTG inducible conditional expression vectors with single (left) and double (right) *lac* operator.
(TIF)

S2 Fig Schematic of the genomic organization in the wild-type and the conditional expression strains generated through single cross over recombination. Typical genomic organization of wild-type (WT) strain and conditional expression strain generated through single cross over recombination. a, b, c, d,e indicate the positions of primers used for screening of right recombinant. Inset: a sample picture of agarose gel electrophoresis of the PCR screen performed. dnstream: downstream, pr: promoter, tr- truncated, fl: full length, hyg^R^: hygromycin resistance gene, KD: knockdown, WT: wild-type.
(TIF)

S1 Table List of primers used in this study. (DOCX)
