# Supplementary figures and images for: An IPTG Inducible Conditional Expression System for Mycobacteria
Source: PLoS One. 2015 Aug 6;10(8):e0134562. doi: 10.1371/journal.pone.0134562 (PMC4527713; doi:10.1371/journal.pone.0134562)

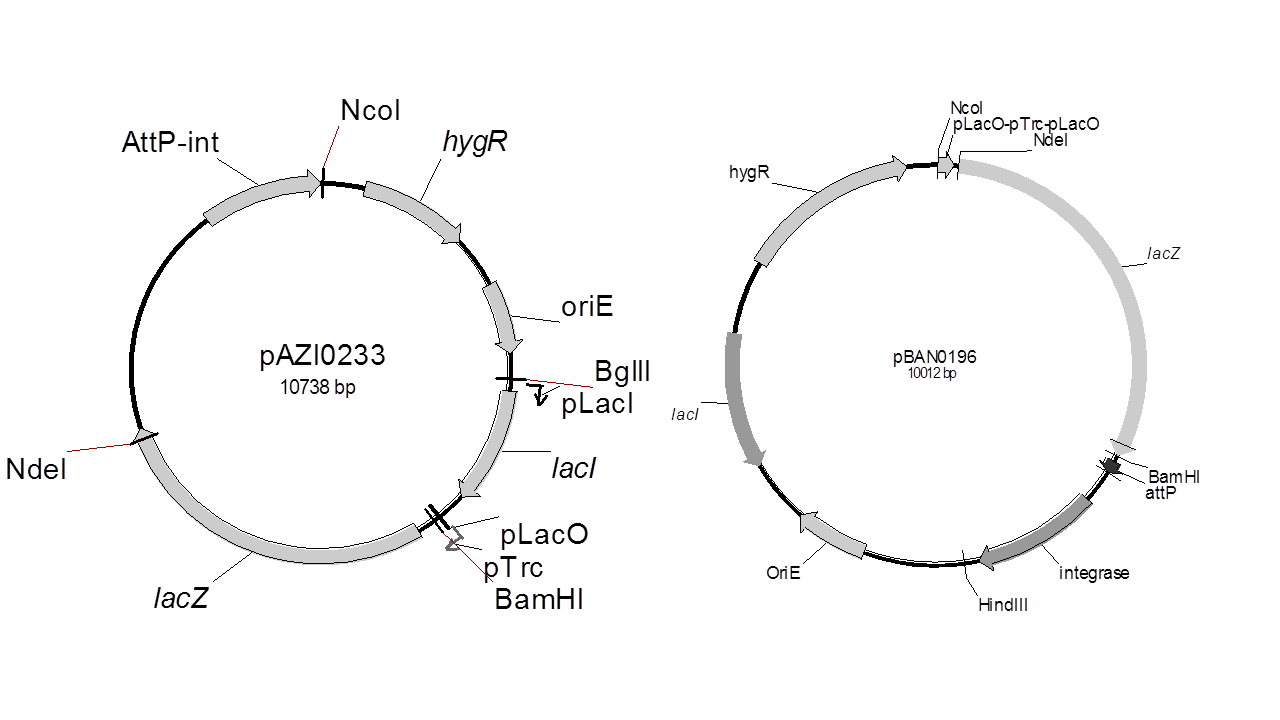

Supplement: S1 Fig — Plasmids with lacZ and attP-int sequences used for the evaluation of regulation of expression from the IPTG inducible conditional expression vectors with single (left) and double (right) lac operator. (TIF) [file pone.0134562.s001.tif]

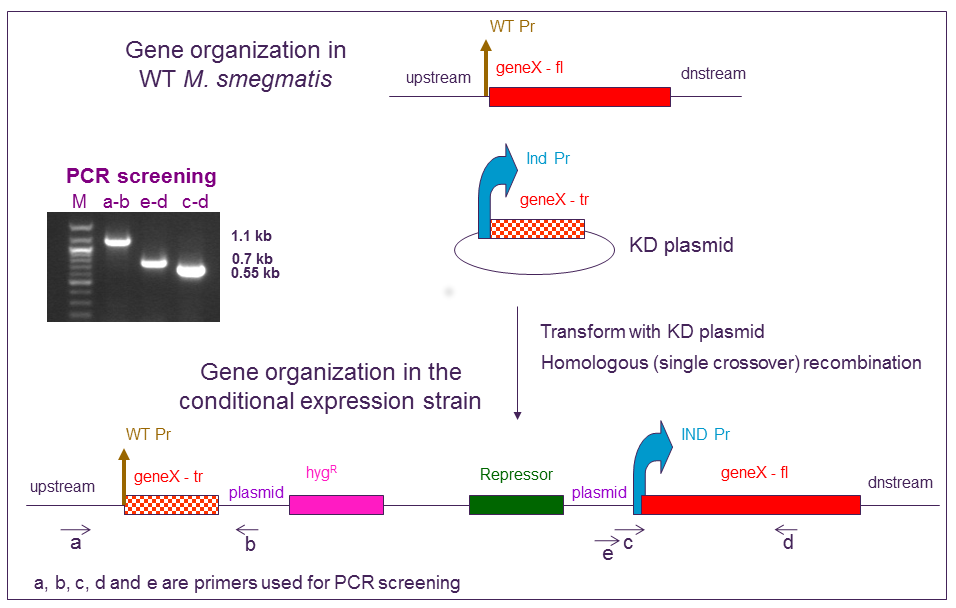

Supplement: S2 Fig — Typical genomic organization of wild-type (WT) strain and conditional expression strain generated through single cross over recombination. a, b, c, d,e indicate the positions of primers used for screening of right recombinant. Inset: a sample picture of agarose gel electrophoresis of the PCR screen performed. dnstream: downstream, pr: promoter, tr- truncated, fl: full length, hygR: hygromycin resistance gene, KD: knockdown, WT: wild-type. (TIF) [file pone.0134562.s002.tif]
