# Supplementary material for: An IPTG Inducible Conditional Expression System for Mycobacteria
Source: PLoS One. 2015 Aug 6;10(8):e0134562. doi: 10.1371/journal.pone.0134562 (PMC4527713; doi:10.1371/journal.pone.0134562)
Supplement: S1 Table — (DOCX) [file pone.0134562.s003.docx]

**Supporting information:**

**S1 Table:**

**List of primers used in this study**

| **Primer name** | **Primer sequence (5’……3’)** | **Details** |
| --- | --- | --- |
| *lacZ*_1F | ATGACCATGATTACGGATTCACTG | Forward PCR primer for *lacZ* |
| *lacZ*_1R | TTATTTTTGACACCAGACCAACTGG | Reverse PCR primer for *lacZ* |

| SynPro | CCATGGGGAATTGTGAGCGCTCACAATTCCATCCAAGAACAACCATCCTAGCAACACGGCGGTCCCCGATTGACAATTAATCATCCGGCTCGTATAATGGAATTGTGAGCGCTCACAATTCCTCTAGA | Synthetic gene with Ptrc promoter and two lac operators |
| --- | --- | --- |
| *lacZ*_Scrn4F | CCTTATTTATCAGCCGGAAAACCTAC | Screening primer for *lacZ* |
| *inhA*_9452F | TCATGCATATGACAGGCCTACTCGAAGGC | Forward primer to amplify *M. smegmatis inhA* |

| *inhA*_9452R | TATAAGCTTACATCGCCAGCGTGCGGATC | Reverse primer to amplify *M. smegmatis inhA* |
| --- | --- | --- |
| *rpoB*_9452F | GTCATCCATATGCTGGAAGGATGCATCTTG | Forward primer to amplify *M. smegmatis rpoB* |
| *rpoB*_9452R | AACGAACGTTACACCTTGACGCTCTGCAGC | Reverse primer to amplify *M. smegmatis rpoB* |
| *ftsZ*_9452F | TACTATCATATGACCCCCCCGCATAACTAC | Forward primer to amplify *M. smegmatis ftsZ* |
| *ftsZ*_9452R | AGATAAGCTTATGTGGTGATCAGGTCGGTG | Reverse primer to amplify *M. smegmatis ftsZ* |
| 9452F1 | TTTTGTTTAACTTTAAGAAGGAGATAT | SCO screening primer |
| 9452F2 | ACAACCATCCTAGCAACACGGCG | SCO screening primer |
| 9452R1 | GAACCTGCAATTAGCCCTTAGGA | SCO screening primer |
| 9452_1F | GTGAGCGCTCACAATTCCTCTAGA | SCO screening primer |
| 9452_1R | CCCTTAGGAGGGGCTCGTTATATCC | SCO screening primer |
| rpoB_F3 | TGCCGCATCCGAATAGTCGCATGAG | *rpoB* SCO screening primer |
| *rpoB*_R4 | GGTGTCGACATTGTCGACGTCGAAC | *rpoB* SCO screening primer |
| *inhA*_F3 | TTTCTCGCTCACAAGGCGTCACCAA | *inhA* SCO screening primer |
| *inhA*_R4 | TCGCCCAGCGCACCGCCCACGATTG | *inhA* SCO screening primer |
| *ftsZ*_F3 | ACCCAACCAGGGAGGAAGACGATCC | *ftsZ* SCO screening primer |
| *ftsZ*_R4 | TCGGACAAGTCCACGTTGATCAGAC | *ftsZ* SCO screening primer |

| *kan*^R^F | ATGAGCCATATTCAACGGGAAACG | Screening primer for kanamycin resistance gene |
| --- | --- | --- |

| *kan*^R^R | GAAAAACTCATCGAGCATCAAATGAAA | Screening primer for kanamycin resistance gene |
| --- | --- | --- |
| *hemHF* | ATTCGGATCCAGTGTCGTTTGACGCCTTGC | Forward PCR primer to clone *hemH* in pAZI272 |
| *hemHR* | TATAGAATTCTCACGCCGAGCAGGCCGG | Reverse PCRprimer to  clone *hemH* in pAZI272 |
| *inh*AF | ATCTGGATCCAATGACAGGCCTACTCG | Forward primer to clone *inhA* in pMV261 vector |
| *inh*AR | GACTAAGCTTTCACAAGAGCTGCGTGC | Reverse primer to clone *inhA* in pMV261 vector |

**S1 Figure:**

**Schematic of the genomic organization in the wild-type and the conditional expression strains generated through single cross over recombination.**

**
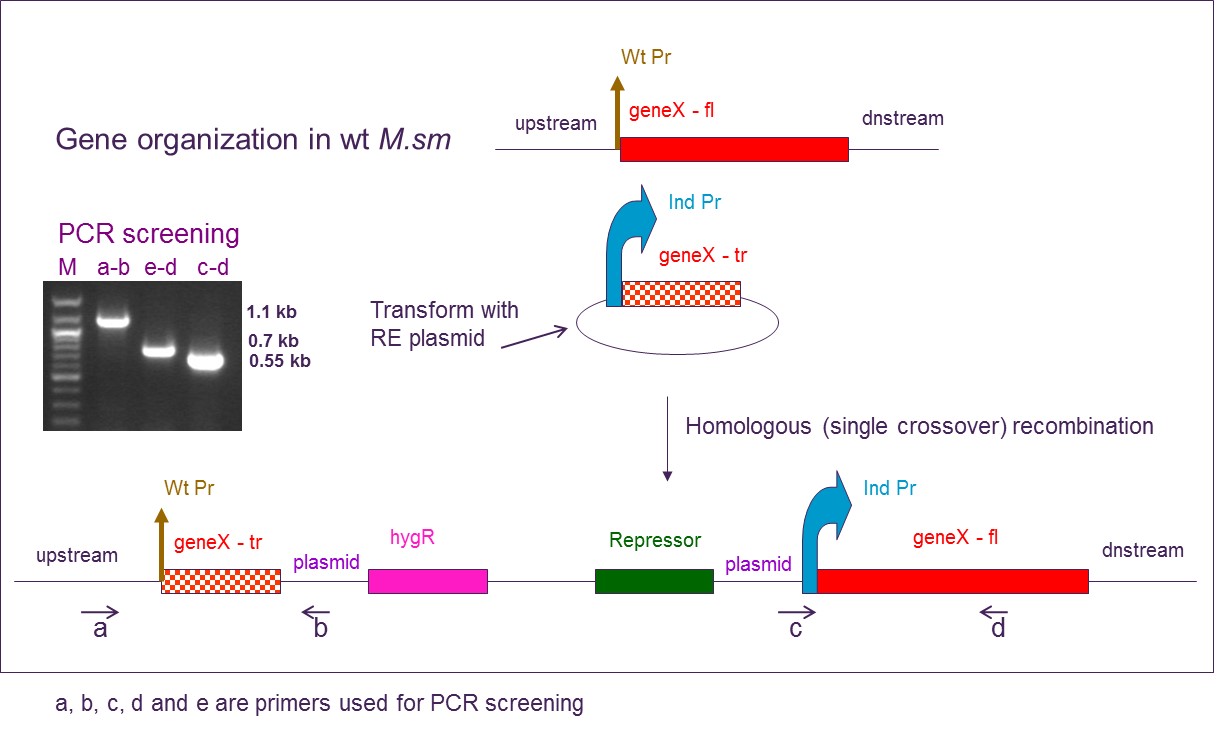
**
